# The crosstalk of intratumor bacteria and the tumor

**DOI:** 10.3389/fcimb.2023.1273254

**Published:** 2024-01-03

**Authors:** Jiating Huang, Yuqin Mao, Lishun Wang

**Affiliations:** ^1^ Center for Traditional Chinese Medicine and Gut Microbiota, Minhang Hospital, Fudan University, Shanghai, China; ^2^ Institute of Fudan-Minhang Academic Health System, Minhang Hospital, Fudan University, Shanghai, China

**Keywords:** intratumor, bacteria, tumor, immune, therapy

## Abstract

The in-depth studies reveal the interaction between the host and commensal microbiomes. Symbiotic bacteria influence in tumor initiation, progression, and response to treatment. Recently, intratumor bacteria have been a burgeoning research field. The tumor microenvironment is under vascular hyperplasia, aerobic glycolysis, hypoxia, and immunosuppression. It might be attractive for bacterial growth and proliferation. As a component of the tumor microenvironment, intratumor bacteria influence tumor growth and metastasis, as well as the efficacy of anti-tumor therapies. Therefore, understanding the intricate interplay of intratumoral bacteria and the host might contribute to better approaches to treat tumors. In this review, we summarize current evidence about roles of intratumor bacteria in tumor initiation and anti-tumor therapy, and what is remained to be solved in this field.

## Introduction

Cancer is a major public health issue worldwide and ranks the second leading cause of death in the United States (Siegel et al., 2023). Even though cancer death rate continues to decline in recent years and the novel anti-tumor immunotherapy is rapidly developing, the response to treatment, such as immune checkpoint inhibitors (ICIs) is still not satisfactory. To be noticed, for most types of cancer, only a minority of patients respond to the ICI treatments. It is reported that the response rate to single agent PD-1 blockade in patients ranges from 40% to 70% in some tumor types, such as melanoma, while most other patients benefit from ICIs are limited to the rates of 10-25% (Schoenfeld and Hellmann, 2020). And unfortunately, many patients with initial response will later develop acquired resistance, with limited understanding of the underlying causes (Schoenfeld and Hellmann, 2020). Accordingly, the remaining challenges are seeking novel preventions for tumorigenesis and approaches to enhancing the unsatisfying response to anti-tumor therapies, especially ICIs. Therefore, it is particularly important to further explore the factors and mechanisms that affect the initiation and progression of tumors.

Commensal flora confers profound impacts on the physical health of human host, including the prevention and therapeutic response to cancer (Tanoue et al., 2019; Zegarra-Ruiz et al., 2021). Numerous studies have revealed that gut microbiota exerts great effects to the initiation and treatment of tumors. Oral administration of some bacteria, such as *Bifidobacterium*, *Lactobacillus*, or *Akkermansia muciniphila* (*A. muciniphila*), can directly suppress tumor growth (He et al., 2021; Bell et al., 2022; Mao et al., 2023), as well as strengthen the therapeutic effects of anti-cancer treatments (Routy et al., 2018; Lee et al., 2021; Zhang et al., 2022). For example, Bell et al. reveals that *Lactobacillus reuteri* suppress the colon cancer growth, by inducing oxidative stress and inhibiting protein translation that tumor progression required (Bell et al., 2022). Routy et al. also illustrates that patients with primary resistance to ICIs have downregulated abundance of *A. muciniphila* (Routy et al., 2018). Fecal microbiota transplantation from response and non-response cancer patients can reproduce the phenotypes on mice. And oral supplementation with *A. muciniphila* with non-responder feces restored the response to PD-1 blockade in tumor mice model (Routy et al., 2018).

Additionally, some pathogens, such as *Fusobacterium nucleatum* (*F. nucleatum*) in the gut, can produce genotoxins that damage the epithelium cell DNA, and induce carcinogenesis (Kostic et al., 2013; Wang and Fang, 2023). Great functions of gut microbiota have been identified on oncogenesis. However, recently, researchers have surprisingly detected and characterized the rich and diverse bacteria located within the tumor tissues (Park et al., 2022). Due to the advent sequencing techniques, it is believed that intratumoral bacteria, as a component of tumor microenvironment, is prevalent in different types of tumors (Nejman et al., 2020). And more and more studies have been dedicated to uncover the role of intratumor bacteria for the host. Microbiome within the pancreatic cancer regions promotes tumor progression by induction of a tolerogenic immune microenvironment, which leads to T-cell anergy (Pushalkar et al., 2018). Accordingly, despite the limited technological advances and understandings, it is compelling that intratumoral bacteria as a component of the tumor microenvironment, play an important role in the tumor initiation, progression, and response efficacy to anti-tumor therapies (Johnston and Bullman, 2022). Here, we outline the effects of the intratumoral bacteria on tumor and summarize the limitations and prospects in this field, which are needed to be solved in the near future.

## The detection of the diversity of intratumoral bacteria

With the development of 16S rRNA sequencing and metagenomics sequencing techniques, amounts of bacterial genomes have been detected in various tumor tissues in recent years. The bacteria detected within the tumor are closely related to the pathological characteristics and therapeutic effects of tumors (**Table 1**) (Johnston et al., 2019). In 2020, Poore et al. analyze the structures of intratumoral microbiota within more than 30 types of tumors and proposed a new cancer diagnostic tool based on the microbiota profile (Poore et al., 2020). Nejman et al. characterize the tumor microbiome on more than 1,500 samples of seven tumor types, including breast cancer, lung cancer, melanoma, and pancreatic cancer by16S rRNA sequencing and immunofluorescence analysis (Nejman et al., 2020). And they find that the intratumor bacteria are primarily intracellular, located in both cancer and immune cells. Different types of tumors harbor specific structures of intratumoral bacteria (Nejman et al., 2020). In addition, the microbiota of breast tumor tissues is the richest and most diverse, in comparison with that in other types of tumors. This study also reveals the association of metabolic functions by intratumoral bacteria and the tumor subtypes. It suggests the predictive functions of tissue-resident bacteria in tumors. The bacteria composition detected in the metastases is closely correlated with that in the primary region, which might indicate the same origin of intratumor bacteria from the primary region to the metastases (Bullman et al., 2017; Fu et al., 2022).

**Table 1:** Characteristics of different types of tumors.

Tumor types	Research	Differential intratumoral bacteria
Glioblastoma multiforme	*Nejman D et al. (Nejman et al., 2020)*	*Acinetobbacter US_424; Neisseria macacae; Enterobacter cloacae*
Bone		*Sphingomonas yanoikuyae; Actinomyces massiliensis; Pseudomonas argentinensis; Enterobacter asburiae*
Ovary		*Roseomonas mucosa; Sphinogomonas US_602; Staphulococcus cohnii*
Pancreas		*Enterobacter asburiae; Klebsiella pneumoniae; Citrobacter freundii; Fusobacterium nucleatum*
Melanoma		*Paracoccus marcusii; Staphylococcus aureus*
Lung		*Sphingomonas yunnanensis; Paracpccus marcusii; Klebsiella pneumoniae; Roseomonas mucosa; Citrobacter freundii*
Breast		*Streptococcus infantis; Lactobacillus iners; Fusobacterium nucleatum; Corynebacterium US_1715; Paracoccus marcusii; Staphylococcus cohnii;etc*
Gastrointestine	Poore GD et al.Poore et al., 2020	*Fusobacterium* spp.
CESC		*Alphopapillomavirus*
COAD		*Faecalibacterium*
LIHC		*Orthohepadnavirus*
Colonrectal cancer	Bullman S et al.Bullman et al., 2017	*Fusobacterium.nucleatum;* *Bacteroides; Selenomonas;* *Prevotella*

TCGA Abbreviation:

CESC, Cervical Squamous Cell Carcinoma and Endocervical Adenocarcinoma; COAD, Colon Adenocarcinoma; LIHC, Liver Hepatocellular Carcinoma.

In addition, the colonization of *F. nucleatum* and *Bacteroides*, *Selenomonas*, and *Prevotella* detected in colorectal cancer also maintained in distal metastasis, suggesting the close association of intratumor bacteria between primary and metastatic legions (Bullman et al., 2017). On the other hand, Galeano et al. demonstrates the distribution of intratumoral bacteria within tumor tissues is specifically accumulated in the lesion with less vascularized, higher immune-suppressive and lower levels of proliferative signal Ki67 expression of tumor cells, as compared with non-detected bacteria tumor regions, by *in situ* spatial-profiling technologies and single-cell RNA sequencing (Galeano Niño et al., 2022).

## The intratumor bacteria promotes the initiation of tumor

As the tumor was considered sterile originally, researchers have begun to uncover the function of intratumoral microbiota on tumor after the detection of intratumor bacteria since the recent years. Intratumoral flora affects the initiation and progression of tumors through a variety of mechanisms, including DNA damage, activation of carcinogenic pathways, induction of immunosuppression, enhancing the resistance of tumor cells against the stress from the host, and metabolizing therapeutic drugs into malfunctioning form (Geller et al., 2017; Dejea et al., 2018; Pushalkar et al., 2018; Pleguezuelos-Manzano et al., 2020).

For example, colibactin­producing *Escherichia coli* can synthesize and secrete colibactin, which directly leads to the cross-linking of host cells in the process of DNA replication, resulting in abnormal cell cycle and carcinogenesis, thus inducing the generation of tumors (Pleguezuelos-Manzano et al., 2020). In addition, *F. nucleatum*, the known oral anaerobic bacterium, contributes to colorectal tumorigenesis. It can migrate from the oral cavity to other regions through digestive tract as well as systemic circulation and function in other niches. *F. nucleatum* colonizes within colorectal tumor regions by binding lectin Fap2 to Gal-GalNAc-overexpressing CRC cells (Abed et al., 2016). Gur et al. demonstrate that different strains of *F. nucleatum* can directly engage with the immune system of the host as well. The Fap2 protein of *F. nucleatum* can inhibit the immune cell activity to protect the tumor cells via binding to human inhibitory receptor TIGIT on NK cells (Gur et al., 2015). Furthermore, *F. nucleatum* can induce a drastic reduction of METTL3-mediated m6A modification to induce CRC aggressiveness and contribute to metastasis (Chen et al., 2022).

Pushalkar et al. reported that pancreatic cancer regions harbor more abundant microbiome, which promotes oncogenesis by induction of a tolerogenic immune microenvironment by differentially activating select toll-like receptors (Pushalkar et al., 2018). Accordingly, ablation of intratumoral bacteria promotes adaptive immune cell differentiation. On the other hand, the intratumor bacteria can also enhance the survival of circulation tumor cells to promote metastatic colonization (Fu et al., 2022). It is reported that in a murine spontaneous breast-tumor model MMTV-PyMT, depletion of intratumoral bacteria fails to suppress the primary tumor growth, but greatly inhibit the lung metastasis. It is because intratumoral bacteria carried by circulating tumor cells strengthen the cellular resistance to fluid shear stress via the reconstruction of actin cytoskeleton (Fu et al., 2022).

## The intratumor bacteria suppresses the tumor growth

However, the intratumor bacteria might not necessarily play a promotive role in tumors. Recently, Wang et al. have found that *Ruminococcus* and *Brautella*, which are symbiotic bacteria of *Spirillaceae* in colon tissue, can promote the function of CD8^+^T cell tumors in immune surveillance, to inhibit the initiation and development of CRC (Zhang et al., 2023). Studies have also found that polypeptides from intratumor bacteria can be presented by tumor cells to activate the immune response, suggesting that intratumor bacteria may be able to activate the immune system by influencing antigen presentation, thus affecting the therapeutic effects (Kalaora et al., 2021). What’s more, Bender et al. demonstrates that merely daily oral administration of frequently used probiotic bacteria strain, *Lactobacillus reuteri*, can efficiently restrain melanoma growth and promote survival (Bender et al., 2023).

## The intratumor bacteria affects the anti-tumor treatment

In addition, intratumoral bacteria can affect the efficacy of antitumor therapy through multiple mechanisms (Xie et al., 2022). Firstly, the intratumor bacteria may cause drug resistance by promoting the metabolism of the anticancer drug into its inactive form. Geller et al. found that *Gammaproteobacterium* within colon cancer contain cytidine deaminase, which can transform the chemotherapy drug, gemcitabine into an inactive form, thus impairing the efficacy to chemotherapy (Geller et al., 2017).

In addition, some studies have found that *F. nucleatum*, as a common intratumoral bacteria that promoting tumor, can also activate the NF-kB signal by activating the cGAS-STING pathway and promoting the expression of PD-L1 in tumor cells, thus enhancing the efficacy of PD-L1 immunotherapy (Gao et al., 2021). Additionally, it is found that *Bifidobacterium* enhance the local anti-CD47 immunotherapy on tumor regions, through its accumulation within the tumor microenvironment (Shi et al., 2020). As the bacterial accumulation in tumor microenvironment, the regional immune response is activated in a stimulator od interferon genes (STING)-and interferon-dependent fashion. The systemic supplementation of *Bifidobacterium* starts its migration to tumor, and the response to anti-CD47 immunotherapy in mice is improved (Shi et al., 2020). Recent studies have found that tumor immunotherapy can promote the migration of intestinal flora to the secondary lymphatic system and tumor lesions, and bacteria can activate DC cells and cytotoxic CD8^+^T lymphocytes (CTLs), thus enhancing the anti-tumor effect of immunotherapy (Choi et al., 2023). Many studies have reported that oral probiotics can regulate the structure of intestinal flora, and play a role in inhibiting tumor growth by producing specific metabolites or regulating the host’s immunity. To be noticed, *Lactobacillus reuteri* by oral gavage can migrate to tumor legions and releases indole-3-aldehyde (I3A) to improve ICI therapy efficacy. It results from the I3A can activate aryl hydrocarbon receptor-dependent CREB and facilitate effector cell functions (Bender et al., 2023).

## The predictive potentials of intratumoral bacteria as biomarkers

Considering the specificity of intratumoral bacteria diversity in various types of tumors (Nejman et al., 2020), as well as its effects on the prevention and treatment of cancer (Pushalkar et al., 2018), the intratumor bacteria might serve as biomarkers to predict the outcomes of patients in clinical practice. For example, it is known that pancreatic ductal adenocarcinoma (PDAC) is one of the most lethal cancers, due to the delayed detection and diagnosis, to some extents (Siegel et al., 2023). Novel predictive biomarkers with sensitivity might help to solve this dilemma. The cancerous pancreas tissues harbor a richer microbiota profile, compared to normal pancreas (Pushalkar et al., 2018). It is found that higher diversity in the profile of intratumor bacteria is detected in patients with longer survival rates, with the specific bacteria cluster, *Pseudoxanthomonas*-*Streptomyces*-*Saccharopolyspora*-*Bacillus clausii* (Riquelme et al., 2019). Additionally, it is also reported that the infiltration of immune cells is positively associated with the diversity of intratumor bacteria (Riquelme et al., 2019). Therefore, structures of intratumor bacteria might be the potential biomarker for therapeutic response and novel target of anti-cancer treatment. Collectively, it suggests that manipulation of intratumor bacteria might be also a novel way to affect tumor immunity and the response to immunotherapy, after better understanding of the interplay of the microbiome and the immune system of the host.

## The source of intratumor bacteria

It is known that the tumor microenvironment is characterized with vascular hyperplasia, aerobic glycolysis, hypoxia, and immune-suppression. Due to hyperplasia and tumor necrosis, the tumor region is in a highly hypoxic and nutrient-rich state that can attract and support the colonization and growth of facultative and/or anaerobic bacteria (Rahma and Hodi, 2019; Nejman et al., 2020). Therefore, the tumor legion is a suitable environment for the bacteria to survive and function in. Notably, due to the leakage of immature blood vessels in tumors, the commensal bacteria migrate from other niches through blood circulation tend to invade and sustain in the tumor region, and might be more likely to escape from the elimination by the immune system of the host, due to the immune-suppressive environment (Jin and Jin, 2020). In addition, the local microenvironment of the solid tumor was in a state of low oxygen, which was conducive to the survival and accumulation of anaerobes or facultative anaerobes (Heymann et al., 2021). Moreover, the necrotic tissue at the tumor site also provides a nutritional environment for bacterial proliferation (Park et al., 2022). However, although the importance of the intratumor bacteria has been realized, the source of these bacteria in the tumor lesions remains unclear. It is noteworthy that the study showed that the composition of bacteria in the tissues varied among different tumor types, with breast cancer harboring the highest bacterial diversity in seven tumor types, including lung cancer and ovarian cancer. The composition and quantity of bacteria in the tumor are tumor-type specific, which suggests the different sources of bacteria in specific tumor lesions. And the microorganisms in the tumor may play different roles in specific tumor environments (Nejman et al., 2020).

## The limitation and prospects of tumor-resident bacteria research

Due to the close crosstalk of commensal bacteria with the host (Zheng et al., 2020), especially the gut bacteria, it is difficult to distinguish the effects of the tumor-resident bacteria from the gut microbiome on the host. Fu et al. selectively eliminates the intratumoral bacteria without influencing the gut flora, by developing a novel tool of choosing specific antibiotics cocktail and administration routes (Fu et al., 2022). Even though the modification of routes or types to antibiotics administration (such as, by intravenously) has enabled to specifically focus on the effects of intratumoral bacteria ablation on the tumor, it still remains largely unknown about the own role of tumor-resident bacteria in tumor growth. In particular, as the antibiotics also clear the beneficial bacteria which might potentially affect the tumor growth, it might hinder to illustrate the real function of select bacteria species in the research. Hence, it would be significant to develop approaches to the precise elimination for selective bacteria, such as phage therapy, to mostly exclude other side effects. Moreover, the majority of bacteria detected within tumor tissues fail to be cultivated, which hinder researches on the functional and molecular mechanisms of the specific bacteria strains to illustrate the roles of intratumoral bacteria. In this regard, it requires more appropriate and advent tools to isolate the intratumoral microbiota and mimic the tumor microenvironment *in vitro*, which would further the mechanism research. As for prospects for the applications of tumor-resident bacteria, it is promising to utilize the bacteria as the adjuvant to improve the anti-tumor therapy, via bacteria-associated-metabolism and immune response in the tumor microenvironment. Tumor colonization with L-Arg bacteria synergizes with PD-L1 blockade to suppress the tumor growth (Canale et al., 2021). As the study reveals that antigens derived from intracellular bacteria are presented by the human tumor cells and elicit immune reactivity, it suggests that bacterial peptides might act as immune activators to elicit the tumor infiltrating T cell responses to therapy (Kalaora et al., 2021). Noticeably, in that study, recurrent bacterial peptides in tumors from different patients are identified, as well as in different tumors from the same patient, which suggests that the bacterial peptides might be possibly universal adjuvants for different patients (Kalaora et al., 2021). And it deserves further exploration to develop the specific bacterial peptides as general biomarkers for prediction in therapy responses.

## Discussions

**Figure 1:**
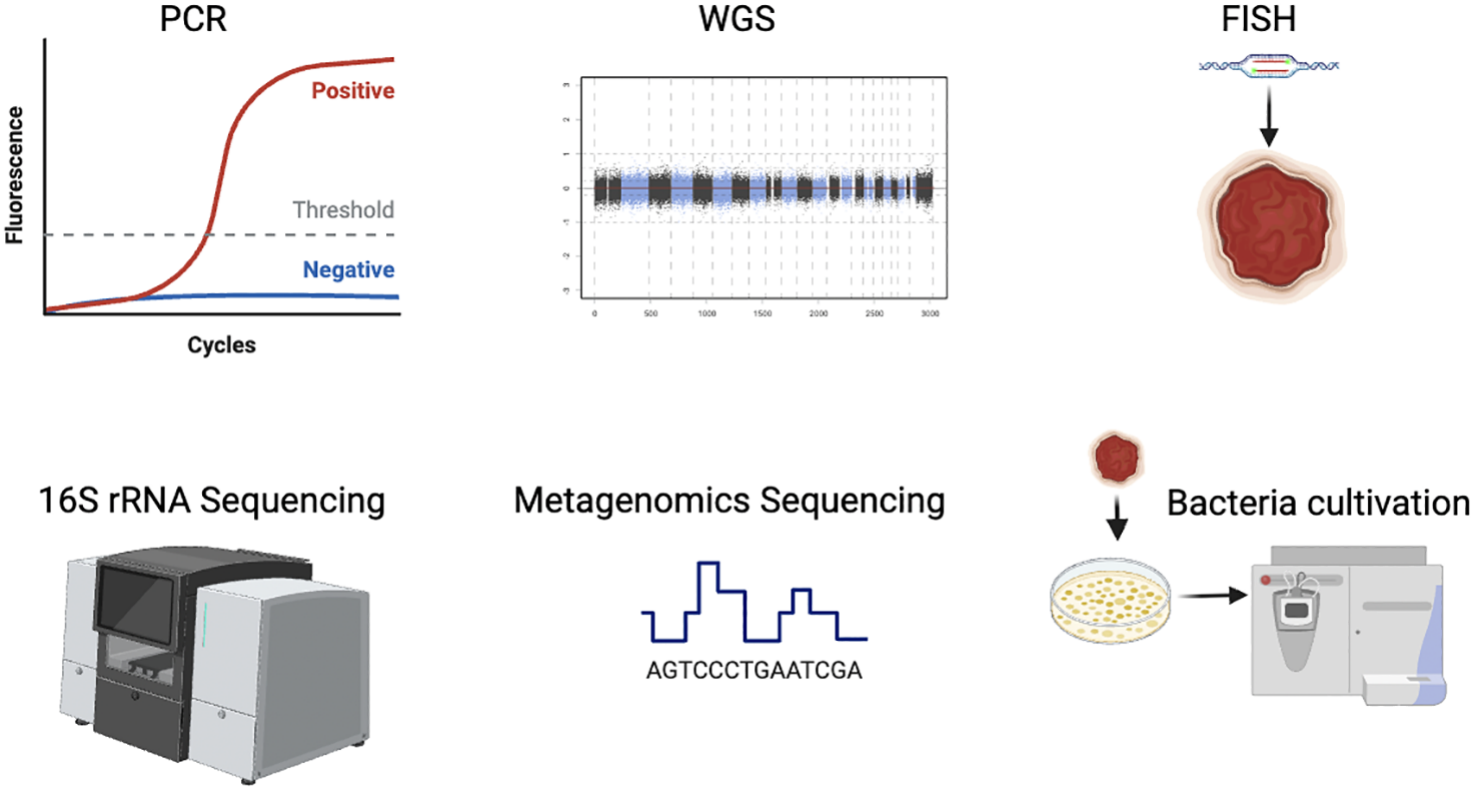
Overview of common methods in intratumoral bacteria research.

Driven by the advent of next-generation sequencing technologies including 16S rRNA or metagenomic sequencing, the tumor-resident bacteria research has been a burgeoning field. They are effective to assess the composition and quantity of bacteria in tumors, by evaluating the abundances of the tumor-resident microbiome. And more compelling studies have demonstrated the universal presence and specific effects of the intratumoral bacteria (Park et al., 2022). In fact, tumor samples have a low bacterial load and contain a large amount of host DNA in the tissues. In the process of nucleic acid extraction, abundant non-bacterial DNA will be mixed, which requires a process of microbial genome enrichment. Additionally, there may be environmental and other exogenous DNA contamination in sampling and processing. Exogenous bacterial DNA may affect the results of sequencing if the original bacterial load of the samples is not ample enough. It is the advent of next-generation sequencing methods, including WGS and metagenomics sequencing, that allow to characterize and analyze comprehensively for the tumor-resident bacteria, despite failure in bacterial isolation and cultivation. However, despite the advances in sequencing techniques, it still remains large to be improved in the precise detection and analysis of the intratumoral bacteria data. To better overview the development of various technologies nowadays, we introduce the common and useful methods in intratumoral bacteria in **Figure 1**.

Besides sequencing, bacterial culture using grinding tissue samples is also an effective method to study intratumor bacteria (**Figure 1**). However, numerous bacteria in tumor tissue are uncultivable *in vitro* due to the rigorous nutrient and survival requirements of bacteria, which is still a major challenge in the illustration of functions od intratumor bacteria. Therefore, the researchers are confronted with the gaps of successful detection but fail to obtain the selective bacteria indicated by sequencing data by bacteria isolation (Johnston and Bullman, 2022).

Given the limitations of *in-vitro* cultivation tools for intratumoral bacteria, the great challenging remains that the intratumor bacteria characterized within specimens lack the validation of their specific functions, with the failure to cultivate *in vitro*. A majority of the current research on intratumoral bacteria is correlational analysis. And since recent years, many studies have reported that bacteria might originate from other niches to the tumor (Bender et al., 2023; Choi et al., 2023). And they act as a vital component in tumor microenvironment, as some of them might crosstalk with the host via their secretion and metabolism to induce an immune-suppressive condition, which promote the development of tumor and help with the bacterial proliferation in the legion (Kostic et al., 2013). it requires further research on the function and mechanism of intratumoral bacteria.

Even though numerous studies increasingly demonstrate the effects of tumor-resident bacteria in tumor progression and metastasis; in fact, unlike in the research on gut microbiota, research of the intratumor bacteria field is still a blue ocean and requires to obtain thorough understandings for roles and potential values of specific bacteria in tumor. The intratumor bacteria might be the novel target to intervene in, which might help to hinder the tumor progress or facilitate the anti-tumor therapy efficacy. A thorough understanding of the tumor microenvironment and the tumor-resident bacteria will open a new chapter for understanding tumorigenesis and cancer therapy (Wang et al., 2023).

## Author contributions

JH: Writing – original draft. YM: Writing – review & editing. LW: Supervision, Writing – review & editing.
